# Depression and anxiety among online learning students during the COVID-19 pandemic: a cross-sectional survey in Rio de Janeiro, Brazil

**DOI:** 10.1186/s40359-022-00897-3

**Published:** 2022-08-03

**Authors:** Luísa Pelucio, Pedro Simões, Marcia Cristina Nascimento Dourado, Laiana A. Quagliato, Antonio Egidio Nardi

**Affiliations:** 1 Institute of Psychiatry, Universidade Federal Do Rio de Janeiro (UFRJ), Rio de Janeiro (RJ), Brazil; 2 Departament of Sociology and Political Science, Universidade Federal de Santa Catarina (UFSC), Rio de Janeiro (RJ), Brazil

**Keywords:** Anxiety, Depression, Students, Pandemic, Online learning, Mental health

## Abstract

**Background:**

The COVID-19 pandemic introduced a global need to explore the potential and challenges of online education.

**Objective:**

To evaluate the presence of depression and anxiety in university students and their level of satisfaction with online learning during the period of social isolation caused by the COVID-19 pandemic.

**Method:**

A cross-sectional design was used to evaluate 152 online learning students from six different university courses: Medicine, Psychology, Law, Engineering, Physiotherapy, and Business. The evaluation of the participants was carried out through an online survey in Rio de Janeiro, Brazil. Also, the Hospital Anxiety and Depression Scale was used to assess participants mental health.

**Results:**

Most of the participants reported emotional impact, followed by learning impact, financial impact, social impact, and technological impact, with a significant difference in the presence of depressive symptoms, but no significant difference in anxiety. The participants presented moderate anxiety levels, with no significant differences between genders, and mild levels of depressive symptoms with significant differences between genders. Also, younger students were more anxious than older students. In addition, female students with less social contact presented more depressive symtoms.

**Conclusion:**

From a clinical perspective, the findings provide insights into mental health among university students during the COVID-19 pandemic. These findings may help in the development of effective screening strategies and in the formulation of interventions that improve the mental health of students.

## Background

In March 2020, with COVID-19 multiplying in several countries, the World Health Organization (WHO) declared that the world had reached a pandemic level [1–3]. Online learning made education accessible during the social isolation period as several countries switched to distance learning for all levels of education. Online education is defined as learning and teaching through a primarily electronic medium with the interaction between learners and their educational materials and activities taking place synchronously or asynchronously in a virtual environment [4].

Online education is not a new concept to educators, but the COVID-19 pandemic introduced a global need to explore its potential and opportunities [4]. However, the transition to online learning presents specific difficulties as teaching methodology requires adaptation, with challenges ranging from evaluating the university’s resources to adapting the practical sessions central to technical degrees [5]. Therefore, not every country has the means and resources to adjust to online learning. A study showed that 85% of the institutions in Europe quickly replaced in-person education with online learning, while only 29% of African institutions met online education requirements [6]. Moreover, since the beginning of February 2020, Chinese colleges and universities have used different learning modes, including online learning based on different platforms, to achieve the goal of suspending classes without suspending learning [7]. In Jordan, new recommendations for converting to online teaching in universities were published to mitigate education issues [8]. However, online learning presents challenges to students as it requires time and learning resources, a set of goals, and plans [4]. In Brazil, a group of institutions conducted a series of surveys with 1056 caregivers and 1556 public school students to understand their thoughts and feelings about online learning [8]. The results showed that lack of motivation and difficulties maintaining the online learning routine were the most significant challenges faced by the students [8, 9]. Another study showed difficulties in student engagement, retention rates, and reported perceptions of missing out on traditional classroom experiences [4]. In Lebanon, Fawaz and Samaha (2020) reported that students’ dissatisfaction with online learning might be attributed to a below-average internet service, rendering students unable to attend classes or participate in online exams [10]. Kasse and Balunywa demonstrated that significant structural vulnerabilities such as lack of internet access or technological ineptitude restricted the full‐scale implementation of online learning in Uganda [11].

Many students struggle with psychological problems during their college years. These problems may be even more apparent during the COVID-19 pandemic with the accompanying restrictions and the transition to an online learning environment, but few longitudinal studies have been conducted to date. As part of the World Mental Health International College Student Initiative (WMH-ICS), a study comparing symptoms and identifying stressors concerning depression, anxiety, and suicidality prior to and during the pandemic was conducted among students attending Ulster University in Northern Ireland (NI), and LYIT, in the Republic of Ireland (ROI). Data were collected from first-year students in September 2019, with a completed response rate of 25.22% (NI) and 41.9% (ROI) to the number of first-year students registered. A follow-up study was conducted in Autumn 2020, with 884 students fully completing the online survey in both years, equating to just under half of those who completed the initial survey. High levels of mental health problems were found in year 1, especially in the ROI. Levels of depression increased significantly in year 2, particularly among students in NI, although anxiety levels decreased. No significant variations were found for suicidal behavior. Several stressors were identified, including increased social isolation and worrying about loved ones [12].

The pandemic caused by COVID-19 also increased depressive and anxiety symptoms and psychological pressures in the general population [10, 12]. A current report suggests that an increased level of depression, stress, and anxiety was found in people who were single, separated, or widowed, lost jobs, or were in contact with potential COVID-19 patients. In addition, people with higher levels of education presented higher stress levels [13]. A meta-analysis [14] reported that the prevalence of depression could be affected by changes in psychiatric practices and the availability of online information on mental health. Another study showed post-traumatic stress disorder (PTSD) correlations during the period of social isolation that included religious practice, reason for quarantine/isolation, education level, and being an infection case [14].

Students might be severely affected by the COVID-19 pandemic with significant impacts on academic achievement and social life. In addition, the discrepancies and inequalities observed at global and institutional levels may strongly impact individual levels. For example, a study [15] showed that younger, poorer female students with a lack of infrastructure, such as limited internet connectivity, demonstrated higher levels of anxiety. In addition, a report on the experience [16] of medical students in the Philippines described the limitations of online learning on medical skills as they need things to be tangible to practice the clinical eye.

Several infrastructural factors in Brazil, such as the electricity and telecommunication deficit, may be a significant barrier to online learning. In this context, evaluating the impact of online learning on students’ mental health in different cultural backgrounds can provide data to help train and prepare teachers and educational professionals and develop new models of mental health protocols and interventions for the target population. Therefore, this study’s research question focuses on the relationship between depression and anxiety in university students and their level of satisfaction with online learning during the period of social isolation caused by the COVID-19 pandemic. Furthermore, we also aim to understand how depressive or anxiety symptoms might be related to other variables such as type of university course, gender, or age during the online learning period. We hypothesize that the university students will present lower levels of satisfaction with online learning and higher levels of depressive and anxious symptoms related to online learning during the COVID-19 pandemic.

## Method

This is a cross-sectional study that evaluated 152 online learning students in Barra Mansa, Volta Redonda, and Resende in the state of Rio de Janeiro, Brazil. Individuals aged between 18 and 65 years old were included in the study from May 2021 to August 2021. All participants who were willing to respond to the assessment were included. It was estimated that 100% of the university students were online due to the social isolation caused by the COVID-19 pandemic. However, only 65%-70% participated in online classes due to internet access problems or non-detailed personal issues. Participants who were not in social isolation or not in active class/enrollment were excluded. Participants were selected by university lectures, which greatly facilitated access to students. During classes, the lecturers invited the students and sent the survey link to access the full online research. Participants from six different university courses were included: Medicine, Psychology, Law, Engineering, Physiotherapy, and Business.

The Ethics Committee of the Institute of Psychiatry of the Universidade Federal do Rio de Janeiro (Federal University of Rio de Janeiro) (UFRJ) approved the study and all participants signed the informed consent form. This study followed the Declaration of Helsinki.

### Procedures

The participants were evaluated online, through google forms. All eligible participants completed an online assessment using a form collecting sociodemographic data (age, education, current medication) and questions with a Likert scale to understand levels of satisfaction with online learning: (1) What do you think of online learning education? (very poor, poor, regular, good, or very good); (2) Do you feel affected by online learning? (yes/no); and (3) How do you feel affected by online learning? (learning, emotional, financial, social, or technological). The questionnaire used to evaluate levels of satisfaction with online learning was developed in Brazilian Portuguese by the authors. In addition, the participants’ anxiety and depression status were also assessed using the Brazilian version of the Hospital Anxiety and Depression Scale (HADS). The HADS consists of 14 questions, seven to assess anxiety and seven to assess depression, with each item scored on a scale of 0 to 3, for a total of 21 points for each scale. Cut-off scores: Mild (8 to 10 points); Moderate (11 to 14 points); Severe (15 to 21 points) [17]. Cronbach’s alpha for the HADS is 0.795.

### Statistical analysis

All statistical analyses were performed with SPSS software for Windows version 22.0. A Kolmogorov–Smirnov test was used to verify the normal distribution between variances. Descriptive statistics analyzed the sociodemographic data of the participants (gender, age, university course, online learning impact) and the clinical characteristics (anxiety and depressive symptoms). Chi-Squared was used to compare the distribution of students and university course. Student’s t-test was used to verify the presence of anxiety and depressive symptoms and whether online learning had an impact. The Duncan Multiple Range Test was used to compare a set of sample means with significant minimum amplitude. Linear regression models were performed separately for anxiety and depression and the best models were selected according to the highest explained variance of R squared (R^2^) and the variance inflation factor (VIF) close to 1, for the collinearity in each independent variable. All significance tests were performed at a 2-tailed level considering a significance level of *P* ≤ 0.05.

## Results

### Sociodemographic characteristics

Most of the participants were female (77%, n = 117), with age ranging from 18 to 65 years old: 55% from 18 to 24 years old (n = 84), 23% from 25 to 34 years old (n = 35), 15% from 35 to 44 years old (n = 23), and 6% from 45 to 65 years old (n = 10).

The university students were from 6 different courses: Medicine (2.6% n = 4), Psychology (65% n = 99), Business (3.9% n = 6), Law (16. 4% n = 25), Engineering (7.2% n = 11), and Physiotherapy (4.6% n = 7). The sociodemographic data are shown in Table 1.

**Table 1:** Sociodemographic data

Variable	N	Percentage
*Gender*		
Female	117	77
Male	35	23
*Age*		
18 to 24 years	84	55.3
25 to 34 years	35	23
35 to 44 years	23	15.1
45 to 65 years	10	6.6
*Undergraduate course*		
Psychology	99	65.1
Law	25	16.4
Engineering	11	7.2
Physiotherapy	7	4.6
Medicine	4	2.6
Business	6	3.9

### Students’ clinical evaluation

The sample presented moderate levels of anxiety (M = 11.2 SD 4.72), with no significant differences between genders (*p* = 0.081) and mild levels of depressive symptoms (M = 8.03 SD 4.22) with significant differences between genders (*p* = 0.005). The sample was divided by age group and there was a significant difference in anxiety according to the students’ age (*p* = 0.050), whereby younger students were more anxious than older students, although there was no difference in the presence of depressive symptoms (*p* = 0.145). There was also no significant difference between anxiety (*p* = 0.268) and depressive symptoms (*p* = 0.615) and the type of university course. The data related to anxiety and depressive symptoms are shown in Table 2.

**Table 2:** Level of Anxiety and Depression according to sociodemographic data

		Anxiety		Depression	
Variable	N	M (SD)	*p*-value	M (SD)	*p*-value
*Gender*					
Female	117	10 (5.44)	–	6.29 (3.93)	–
Male	35	11.59 (4.44)	0.081	8.55 (4.18)	0.005
Total:	152				
*Age*					
18 to 24 years	84	12.12 (4.10)		8.7 (3.98)	
25 to 34 years	35	10.43 (4.82)		7.49 (4.49)	
35 to 44 years	23	10.09 (5.93)		6.78 (4.57)	
45 to 65 years	10	9.1 (5.15)	–	7 (3.88)	–
Total	152	11.22 (4. 72)	0.050	8.03 (4.22)	0.145
*Undergraduate course*					
Psychology	99	10.58 (4.98)		7.66 (4.41)	
Law	25	12.84 (3.76)		8.2 (3.04)	
Engineering	11	11.55 (4.43)		8.82 (4.64)	
Physiotherapy	7	13.14 (5.58)		10.29 (6.02)	
Medicine	4	12.75 (2.75)		9.25 (2.63)	
Business	6	11.33 (4.72)	–	8.5 (3.14)	–
Total	152	11.22 (4.72)	0.268	8.03 (4.22)	0.615

*M* mean; *SD *standard deviation

### Online learning levels of satisfaction

Table 3 shows students’ opinions about online learning according to the presence of anxiety and depression. Most of the students considered online learning as regular (34.9% n = 53), followed by good (24.3% n = 37), and poor (23% n = 35). Few students found online learning to be very poor (12.5% n = 19) or very good (5.3% n = 8). There was a significant difference between anxiety (*p* = 0.019) and depressive symptoms (*p* = 0.009) and level of satisfaction with online education. There was also a significant difference between level of satisfaction with online learning and students’ age (*p* = 0.001). Younger students presented more dissatisfaction with online learning than older students (Table 4).

**Table 3:** Students’ opinions about online learning according to the level of anxiety and depressive symptoms

		Anxiety		Depression	
	N	M (SD)	*p*-value	M (SD)	*p*-value
*What do you think of online education?*					
Very poor	19 (12.5)	13.16(3.76)	0.019	10.42 (4.43)	0.009
Poor	35 (23)	12.37 (3.74)	–	8.46 (3.77)	–
Regular	53 (34.9)	10.91 (4.62)		8.23 (4.31)	
*Do you feel affected?*					
Good	37 (24.3)	9.38 (5.35)		6.3 (3.53)	
Very Good	8 (5.3)	12.25 (5.8)		7.12 (5.41)	
Not affected	12(7.9)	9.5(5.56)	0.189	4.83 (3.09)	0.006
Affected	140 (92.1)	11.37 (4.63)	–	8.3 (4.20)	–
*How do you feel affected by online learning?*					
Not affected	11(7.2)	9.82(6.06)	0.069	5.09(3.56)	0.031
Learning	45 (29.6)	11.44(4.43)	–	8.53 (4.17)	–
Emotional	74 (48.7)	11.57 (4.63)		8.34 (4.28)	
Financial	4(2.6)	7.25 (3.5)		9.75 (5.12)	
Social	14(9.2)	9.57 (4.79)		10.5 (3.87)	
Technological	4(2.6)	16(0.81)			

*M *mean; *SD* standard deviation

**Table 4:** Level of satisfaction with online learning by age group

					Chi-Squared
Age	18 to 24 years (%)	25 to 34 year (%)	35 to 44 years (%)	45 to 65 years (%)	0.001
*What do you think of online education?*					
Very poor	89.5	10.5			
Poor	60.0	28.6	5.7	5.7	
Regular	56.6	28.3	15.1		
Good	40.5	16.2	32.4	10.8	
Very Good	12.5	25.0	12.5	50.0	
Total	55.3	23.0	15.1	6.6	

The impact of the pandemic was also investigated (“Do you feel affected by online learning?”). Most students answered yes (92% n = 140), with a significant difference in the presence of depressive symptoms (*p* = 0.006), but no significant difference in anxiety (*p* = 0.189).

The participants were also asked “How do you feel affected?”. Most participants reported emotional impact (48.7% n = 74), followed by learning impact (29.6% n = 45), financial impact (2.6% n = 4), social impact (9.2% n = 14), technological impact (2.6% n = 4), and not affected/none (7.2% n = 11), with a significant difference in the presence of depressive symptoms (*p* = 0.031), but no significant difference in anxiety (*p* = 0.069).

### *Regression models of the factors related to anxiety and depression (*R^2^)

Table 5 shows that students’ anxiety is related to age and financial impact, whereby younger age and more significant financial impact are perceived with increased anxiety (*p* < 0.001). Students’ depression is impacted by gender and social impact, whereby being female and having less social contact result in higher levels of depression (*p* < 0.001).

**Table 5:** Regression models of factors related to anxiety and depression (R^2^)

Predictors	B	b	R^2^	Adj R^2^	Significance
*Anxiety*^a^					
(Constant)	13,290 (0.784)*		.070	.057	.005^a^
Financial Impact	− 4635 (2.331)**	− .158			
Age	− 1124 (0.395)**	− .226			
*Depression*^b^					
(Constant)	8861 (0.397)*		.088	.076	.001^b^
Gender	− 2495 (0.788)**	− .249			
Social Impact	− 2826 (1.148)**	− .194			

^a^Predictors: (Constant), Age, Financial Impact

^b^Predictors: (Constant), Social Impact, Gender

*p < 0.001, **p < 0.05

## Discussion

To the best of our knowledge, this is the first Brazilian study to provide information on university students’ anxiety and depressive levels during the social isolation period. This study aimed to evaluate depression and anxiety in university students and their level of satisfaction with online learning during the period of social isolation caused by the COVID-19 pandemic. The participants presented moderate anxiety levels, with no significant differences between genders, and mild levels of depressive symptoms with significant differences between genders. Also, younger students were more anxious than older students. In addition, female students with less social contact presented higher levels of depression. Our results align with a U.S. nationwide survey [18, 19] among faculty and students in June 2020, which highlighted the gender disparities in online learning during the pandemic, whereby female faculty and students reported more challenges in technological issues and adapting to remote learning compared with their male peers. Another study [20] showed almost half of students presenting anxiety levels ranging from mild to severe, with females reporting higher anxiety scores. Also, Saddick et al. [21], in a large sample of 7,228 university students from Poland, demonstrated a significant increase in depression levels as the pandemic progressed, with female students scoring significantly higher than male students on depression, anxiety, and stress. Similar studies conducted longitudinally among college students found a significant increase in depression and anxiety compared to previous COVID-19 levels [16].

The COVID-19 pandemic has disrupted the lives of all, including university students, especially with the preventive measures to reduce the transmission of virus, leading to all face-to-face teaching and learning being converted to e-learning. The COVID-19 pandemic and the implementation of e-learning may have influenced students’ mental conditions. A study aimed to determine the association of factors with mental health status (depression, anxiety, and stress) among tertiary education students in Malaysia, from both private and public universities, recruited via university emails and social media. The survey was administered via the online REDCap platform, from April to June 2020, during the movement control order period in the country. The questionnaire captured data on socio-demographic characteristics, academic information, implementation of e-learning, perception towards e-learning and COVID-19; as well as DASS 21 to screen for depression, anxiety, and stress. The levels of stress, anxiety and depression were 56.5% (95% CI: 50.7%, 62.1%), 51.3% (95% CI: 45.6%, 57.0%), and 29.4% (95% CI: 24.3%, 34.8%) respectively. Most participants had a good perception of e-learning but a negative perception of COVID-19.

The present study shows that social isolation contributed to depressive symptoms in university students. The impact of the social isolation period on university students may be burdensome due to its perceived effect on their activities of daily living and studies [13]. Fawaz and Samaha (2020) point out that university students are characteristically susceptible to developing stress and depression with an expected increase during the COVID-19 pandemic related to their psychological challenges, conditions in terms of learning, uncertainties about the future, fear of infection, news about lack of personal protective equipment, quarantine induced boredom, frustrations, lack of freedom, and fears caused by rumors and misleading news in the media [10, 20, 21]. Moreover, social isolation may also result in sedentary behavior, which is detrimental to preventing physical, cognitive, psychological, and social health problems [15]. Thus, low self-esteem, feelings of worthlessness, and loss of autonomy may also be related to the presence of levels of anxiety and depressive symptoms found in our study. Further studies should investigate psychological distress to evaluate its impact on depression and anxiety levels in this population.

Our results align with a study that explored the association between the effects of home-based learning during the pandemic and the risks of depression, anxiety, and suicidality among junior and senior high school students. An online survey using the Patient Health Questionnaire (PHQ-9) and Generalized Anxiety Disorder (GAD-7) was conducted between 12 and 30 April 2020, on a total of 39,751 students. Multivariable logistic regression analysis was used to analyze the risk factors of associated depression, anxiety, and suicidality during the pandemic. The prevalence of depression, anxiety symptoms, and suicidality found was 16.3% (95% CI: 16.0, 16.7), 10.3% (95% CI: 10.0, 10.6), and 20.3% (95% CI: 19.9, 20.7), respectively. Female participants and those in junior high school with poor overall sleep quality, poor academic performance, and very worried about being infected during COVID-19 were highly associated with the risk of depression, anxiety symptoms, and suicidal ideation [21]. Another study, conducted via an online survey among 5100 medical students from Wannan Medical College in China, aimed to assess the mental health status of medical students engaged in online learning at home during the pandemic, exploring the potential risk factors for mental health. The Depression, Anxiety and Stress scale (DASS-21) was used to measure self-reported symptoms of depression, anxiety, and stress among 4115 medical students. Nearly one-third of medical students survived with varying degrees of depression, anxiety, and stress symptoms during online learning in the COVID-19 pandemic [22]. These findings demonstrated that the mental status of university students was greatly affected during the COVID-19 pandemic [23].

We also investigated the level of satisfaction with online learning and its impact on students’ lives. We found that students who felt impacted by their financial situation had an increase in their anxiety as demonstrated on the HADS scale, corroborating studies that show the mental and emotional impacts on students’ daily lives [20, 21]. Additionally, we found that most of the students considered online learning as regular, with significant differences between the level of anxiety and depressive symptoms and level of satisfaction with online education. Most of the students reported an emotional impact related to the social isolation period and online learning, with significant differences in depressive symptoms, followed by learning impact, financial impact, social impact, and technological impact. We also found that younger students reported more dissatisfaction with online learning compared to older students. Students’ intentions and attitudes towards online education may play important roles in retention rates and final achievements in online learning. Studies have shown that student interactions have a close relationship with emotional and social engagement and a sense of community, which is significant in effectively promoting learning engagement [21]. We may assume that these students may have to deal with unexpected and continuous changes such as lack of interpersonal contact and daily university activity and the need to adapt to their home routine and resources. However, besides these personal aspects, there is a need to discuss the effectiveness of online learning and its potential barriers in developing contexts. Students from developing countries presented lower scores in online learning and were more likely to withdraw from online courses than their colleagues in developed countries [21].

One of the major challenges in the Brazilian education system is the inequality of educational resources, including usage of computers, internet access, and other technological resources [8]. A survey conducted by a group of institutions in Brazil found that internet access (23%) was the main issue in remote learning, followed by content difficulties (20%), lack of devices (15%), and lack of interest (15%) [16, 22]. Therefore, our results may be related to both students’ intentions and attitudes and the quality of educational resources.

### Strengths and limitations

Our findings can help the development of actions to identify the need for medical and psychological interventions for university students during periods of online learning. For example, universities should incorporate epidemiological practices and involve health professionals as supervisors and counselors throughout the programs [24, 25]. However, our results should be interpreted with caution as this study has several limitations. First, the use of a small convenience sample and its descriptive nature through an online survey with few variables may not allow generalization of the results. Students already diagnosed with depression or anxiety were excluded from the study through an interview prior to the start of testing. Anxiety and depressive symptoms may have been due to many factors other than COVID-19, which may not have been captured through this method. Secondly, the nature of self-reported data in the survey may lead to response biases. This study mainly used self-reported questionnaires to measure psychiatric symptoms and did not make a clinical diagnosis. The gold standard for establishing a psychiatric diagnosis involves a structured clinical interview and functional neuroimaging. In addition, the statistical analysis did not provide evidence of a causal nature. However, our hypotheses were well targeted based on the psychological evidence available in the previous literature [26–29]. Finally, we did not adjust for multiple comparisons, which may bias *P*-values as measures of significance. However, our results are clinically significant as they may provide suggestions for policy makers regarding improving students’ performance and prevent mental health problems.

## Conclusions

Clinically, our findings provide insights into mental health among some university students during the early stages of the COVID-19 pandemic. These findings can be used to better identify students who may struggle during the following stages of the pandemic and in future crises. Our findings can also contribute to the development of effective screening strategies and the formulation of interventions that improve students’ mental health and may even help in the development of strategies to keep students in education.

It is important that students who perceive the need for psychological support can seek professional help to prevent and reduce symptoms.

## Data Availability

The data sets used during the current study can be provided by the corresponding author [L.P], upon reasonable request.
